# Notable transmitted HIV drug resistance among people who inject drugs in Pakistan

**DOI:** 10.1093/jac/dkaf425

**Published:** 2025-11-11

**Authors:** Stephanie Melnychuk, Laura H Thompson, Chris Archibald, James F Blanchard, Faran Emmanuel, Tahira Reza, Nosheen Dar, Paul Sandstrom, Souradet Y Shaw, Marissa L Becker, François Cholette

**Affiliations:** Institute for Global Public Health, College of Community and Global Health, University of Manitoba, Winnipeg, Canada; National Sexually Transmitted and Blood Borne Infections Laboratory, National Microbiology Laboratory at the J.C. Wilt Infectious Diseases Research Centre, Public Health Agency of Canada, Winnipeg, Canada; Sexually Transmitted and Bloodborne Infections Surveillance Division, Centre for Communicable Diseases and Infection Control, Public Health Agency of Canada, Ottawa, Ontario, Canada; Sexually Transmitted and Bloodborne Infections Surveillance Division, Centre for Communicable Diseases and Infection Control, Public Health Agency of Canada, Ottawa, Ontario, Canada; Institute for Global Public Health, College of Community and Global Health, University of Manitoba, Winnipeg, Canada; Institute for Global Public Health, College of Community and Global Health, University of Manitoba, Winnipeg, Canada; Centre for Global Public Health, Pakistan, Chak Shahzad, Islamabad, Pakistan; Canada-Pakistan HIV/AIDS Surveillance Project, Islamabad, Pakistan; National Sexually Transmitted and Blood Borne Infections Laboratory, National Microbiology Laboratory at the J.C. Wilt Infectious Diseases Research Centre, Public Health Agency of Canada, Winnipeg, Canada; Department of Medical Microbiology and Infectious Diseases, Max Rady College of Medicine, University of Manitoba, Winnipeg, Canada; Institute for Global Public Health, College of Community and Global Health, University of Manitoba, Winnipeg, Canada; Department of Medical Microbiology and Infectious Diseases, Max Rady College of Medicine, University of Manitoba, Winnipeg, Canada; Institute for Global Public Health, College of Community and Global Health, University of Manitoba, Winnipeg, Canada; National Sexually Transmitted and Blood Borne Infections Laboratory, National Microbiology Laboratory at the J.C. Wilt Infectious Diseases Research Centre, Public Health Agency of Canada, Winnipeg, Canada; Department of Medical Microbiology and Infectious Diseases, Max Rady College of Medicine, University of Manitoba, Winnipeg, Canada

## Abstract

**Background:**

Transmission of drug-resistant HIV strains to treatment-naïve patients can compromise antiretroviral therapy (ART) effectiveness and lead to treatment failure. In Pakistan, transmitted HIV drug resistance among people who inject drugs (PWID) is fuelled by a lack of harm reduction, ART, poor drug adherence, and unsafe injection practices, resulting in efficient transmission in large injecting networks.

**Methods:**

A cross-sectional study was conducted among PWID recruited in Karachi, Larkana, Peshawar, Quetta and Hyderabad (August to December 2014). A portion of the HIV *pol* gene was amplified from HIV-reactive dried blood spot specimens (*n* = 282/367) and sequenced using an in-house Sanger sequencing assay for HIV drug resistance mutation (DRM) genotyping. DRMs were identified using the Stanford University HIV Drug Resistance Database (https://hivdb.stanford.edu/hivdb).

**Results:**

Overall, HIV subtype A1 was dominant (78.0%; *n* = 220), followed by CRF02_AG (15.6%; *n* = 44), CRF35_AD (2.5% *n* = 7), recombinants (3.5%; *n* = 10), and subtype C (0.4% *n* = 1). DRM analysis identified that over half (63.8%) of participants harboured at least one DRM, of which 28.9% reported using help from a professional injector. Nearly all (99.4%) participants were not actively receiving ART because most (88.7%) had never undergone HIV testing and were unaware of their status.

**Conclusions:**

Findings suggest significant transmitted HIV drug resistance present among PWID, exacerbated by unsafe injection practices, particularly professional injection. Low testing rates signal a need for more comprehensive testing programs to improve HIV status awareness and ART coverage in Pakistan. These gaps remain especially urgent given persistent challenges in the national response, including low ART coverage, poor adherence, and limited access to genotypic resistance testing.

## Introduction

In Pakistan, HIV prevalence was estimated at 0.2% in the general population in 2023, but is substantially higher among people who inject drugs (PWID), with an estimated 38% of PWID living with HIV as of 2016.^1,2^ This considerable burden is attributable to the marginalization of PWID due to contextual factors, including illiteracy, poverty, homelessness, and criminalization,^3–5^ which constrain access to both harm reduction programs and antiretroviral therapy (ART).^6^ Further, the requirement of individuals actively injecting drugs to undergo a two-week drug detoxification before ART initiation and the lack of a national opioid substitution therapy program in Pakistan present structural barriers which might contribute to higher rates of HIV transmission among PWID overall, hindering effective HIV management and posing challenges to overall HIV epidemic control efforts in the country.^7–10^

PWID undergoing ART have increased vulnerability to treatment interruptions due to a lack of social support and stable housing, mental health comorbidities, and effects of stigma and discrimination.^8,11,12^ Suboptimal therapy and non-adherence to ART facilitate the selection of viral strains harbouring drug resistance mutations (DRMs),^13^ which can be transmitted to ART-naïve individuals, constituting what is defined as transmitted drug resistance.^14^ Transmitted drug resistance poses challenges to virological suppression and achieving undetectable viral loads in ART-naïve patients,^15,16^ with studies documenting a high risk of failing first-line therapy in ART-naïve patients harbouring DRMs.^17–19^

The disproportionate burden of transmitted drug resistance facing PWID may be fuelled by the rapid and efficient transmission of drug-resistant HIV strains in large, interconnected injecting networks with a high frequency of sharing injecting equipment in certain contexts,^20,21^ including Pakistan.^22^ High daily injection frequency,^23^ sharing of syringes and other drug paraphernalia,^22^ injection of shared blood-drug mixtures,^24^ and help from professional injectors,^3^ have been previously identified as conditions under which HIV transmission among PWID in Pakistan is more likely to occur.

Few studies have looked at baseline HIV DRMs among people living with HIV in Pakistan,^5,25–27^ with even fewer looking specifically at PWID. Knowledge of DRMs is essential for guiding ART selection, as ARTs are currently prescribed in Pakistan without baseline drug resistance genotyping.^26^ This increases the risk of ineffective treatment. Using data from a cross-sectional survey conducted in 2014, we sought to assess HIV drug resistance in PWID across five major cities in Pakistan.

## Materials and methods

### Study setting

For this cross-sectional study, PWID were recruited from August to December, 2014, across five major cities in Pakistan. To ensure accurate reflection of the study population, a detailed mapping of the size, geography, and operation typology of the PWID population was conducted prior to the survey as described previously elsewhere.^28^

Following informed consent, participants were interviewed to collect information pertaining to sociodemographic information, drug use practices, and utilization of HIV prevention and care services, as well as a dried blood spot (DBS) specimen.

### HIV serology and *pol* sequencing

DBS were shipped to the National Sexually Transmitted and Blood Borne Infection Laboratory (Public Health Agency of Canada, Winnipeg, Canada). Serological testing using the AVIOQ HIV Microelisa System (Avioq Inc., Durham, NC) was performed according to the manufacturer’s instructions. HIV DRM genotyping was attempted on all HIV-reactive DBS.

A routine, in-house HIV DRM genotyping assay was used to amplify and sequence a portion of the *pol* open reading frame (nt 2074–3334 on HXB2, K03455) from DBS as previously described.^29^ Sequence analysis and contig assembly was performed using RECall.^30^ HIV *pol* sequences are available via a public repository (i.e. NCBI) under accession numbers MN887780-MN888069.

### HIV subtyping and DRM analysis

PWID HIV *pol* sequences were aligned, visually inspected, and manually edited in MEGA v7 as required.^31^ Subtyping was carried out using the REGA HIV automated subtyping tool v3 and COMET,^32,33^ with SCUEAL^34^ used as a tiebreaker for discordant results.

The Stanford HIV Drug Resistance Database program v9.4.1^35^ (https://hivdb.stanford.edu) was used to assess for mutations in the HIV *pol* gene associated with resistance to protease and reverse transcriptase inhibitors. Sequences containing one or more DRMs were classified as being drug-resistant. Any DRMs identified for which no clinical implications were found in the literature were discarded.

### Statistical analyses

Descriptive statistics were calculated for sociodemographic and behavioural information. Pearson’s chi-square test was applied between drug resistance classification and predictor variables using SciPy,^36^ where *P* < 0.050 was used to define statistically significant associations.

### Ethics

Ethics board approval was obtained from the Health Research Ethics Board at the University of Manitoba [HS15691 (H2012:294)] and BRIDGE Consultants Foundation, Pakistan. Informed consent was obtained verbally from study participants and documented by a member of the data collection team for the behavioural survey and biological sampling components of this study. Verbal consent was chosen due to varying levels of literacy among study participants and as an additional measure of protecting their identities. All experiments were conducted in accordance with the Declaration of Helsinki and national and institutional standards.

## Results

### Study population and sequence dataset

A total of 1453 PWID consented to participate in the study from Karachi (*n* = 300), Larkana (*n* = 300), Peshawar (*n* = 253), Quetta (*n* = 300) and Hyderabad (*n* = 300). All participants consented to providing a DBS sample. Serological testing identified 367 (25.5%) PWID living with HIV.

Of the HIV-reactive DBS specimens, 287 *pol* sequences (79.0%) were amplified—corresponding to 80% of HIV-reactive DBS from each city except Hyderabad, where amplification success was closer to 70%. In accordance with the WHO HIV drug resistance criteria,^37^ five sequences containing premature stop codons were removed from subsequent analysis. Of 282 *pol* sequences, 109 (38.7%) were from Karachi, 90 (31.9%) from Peshawar, 39 (13.8%) from Hyderabad, 25 (8.9%) from Larkana, and 19 (6.7%) from Quetta. Most sequences were classified as subtype A1 (*n* = 220, 78.0%), followed by CRF02_AG (*n* = 44, 15.6%), CRF35_AD (*n* = 7, 2.5%), recombinants (*n* = 10, 3.5%), and subtype C (*n* = 1, 0.4%).

### Sociodemographic characteristics

Sociodemographic and behavioural information of PWID for whom HIV sequences were obtained is presented in Table 1. All participants were male. Of 282 sequences analyzed, 180 (63.8%) were classified as drug-resistant.

**Table 1.** Sociodemographic and behavioural characteristics of PWID in Pakistan stratified according to presence of DRMs in HIV sequences

Variable	≥1 DRM (*N* = 180)	No DRMs (*N* = 102)	*P* ^a^
*City, n*			<0.001
Hyderabad	35 (89.7)	4 (10.3)	
Karachi	75 (68.8)	34 (31.2)	
Larkana	22 (88.0)	3 (12.0)	
Peshawar	30 (33.3)	60 (66.7)	
Quetta	18 (94.7)	1 (5.3)	
*Age, n (%)*			0.010
<24	43 (51.8)	40 (48.2)	
25–29	71 (73.2)	26 (26.8)	
≧30	66 (64.7)	36 (35.3)	
*Years injecting drugs, n (%)* ^b^			0.200
<2	32 (60.4)	21 (39.6)	
2–4	64 (59.3)	44 (40.7)	
≧5	84 (70.0)	36 (30.0)	
*Times injecting the previous day, n (%)*			0.130
0	45 (75.0)	15 (25.0)	
1	74 (60.7)	48 (39.3)	
≧2	61 (61.0)	39 (39.0)	
*Injection method at last injection, n (%)*			0.200
Without help	104 (59.8)	70 (40.2)	
Used help, but not from a professional injector	24 (70.6)	10 (29.4)	
Used help from a professional injector	52 (70.3)	22 (29.7)	
*Current ART treatment status, n (%)*			0.720
No	19 (61.3)	12 (38.7)	
Yes	1 (100.0)	0 (0.0)	
Not specified^c^	160 (64.0)	90 (36.0)	

^a^ *P*-values were calculated using Pearson chi-square tests.

^b^Among respondents does not add to 100%.

^c^Low response rate for HIV treatment status is because most participants had never undergone HIV testing and did not respond to questions regarding ART.

Statistically significant predictors of drug resistance classification were city of residence and age (*P* < 0.050) (Table 1). Years injecting, daily injection frequency, method at last injection, and ART status were not significantly associated with drug resistance. Prevalence of drug resistance ranged from 33.3% to 94.7% among participants when stratified by city. Of all sequences containing DRMs, the highest proportion (*n* = 71/180, 39.4%) was between 25 and 29 years of age. Receiving help at last injection, either from a professional or non-professional injector, was common among participants with DRMs (70.3% and 70.6% respectively). Of participants who had undergone HIV testing and were aware of their positive status (*n* = 32, 11.3%), one (3.1%) was actively receiving ART.

### Analysis of HIV drug resistance analysis

HIV mutations associated with drug resistance are summarized in Table 2. Of 282 analyzed HIV *pol* sequences, 13 DRMs were identified among the 180 participants classified as having drug resistance: two protease inhibitor (PI), two nucleoside RT inhibitor (NRTI), and nine non-nucleoside RT inhibitor (NNRTI) mutations (Table 2).

**Table 2.** Drug resistance mutations in HIV sequences from PWID across several Pakistani cities

		Drug resistance/functional association^a^	All cities (*N* *=* 282)	Hyderabad (*N* *=* *39*)	Karachi (*N* *=* 109)	Larkana (*N* *=* 25)	Peshawar (*N* *=* 90)	Quetta (*N* *=* 19)
*DRMs, n (%)*								
*PIs*	L10F	LPV/r, DRV/r	1 (0.4)	0 (0.0)	0 (0.0)	0 (0.0)	1 (1.1)	0 (0.0)
	*M46I	LPV/r, ATV/r	1 (0.4)	0 (0.0)	1 (0.9)	0 (0.0)	0 (0.0)	0 (0.0)
*NRTIs*	*K219R	AZT	2 (0.7)	0 (0.0)	2 (1.8)	0 (0.0)	0 (0.0)	0 (0.0)
	S68G	65R-mediated improved fitness	1 (0.4)	0 (0.0)	0 (0.0)	0 (0.0)	1 (1.1)	0 (0.0)
*NNRTIs*	*K101E	DOR, EFV, ETR, RPV, NVP	2 (0.7)	0 (0.0)	0 (0.0)	0 (0.0)	2 (2.2)	0 (0.0)
	*K103N	EFV, NVP	3 (1.1)	0 (0.0)	0 (0.0)	0 (0.0)	2 (2.2)	1 (5.3)
	V106I	ETR	1 (0.4)	0 (0.0)	0 (0.0)	0 (0.0)	1 (1.1)	0 (0.0)
	E138A	ETR, RPV	164 (58.2)	34 (87.2)	70 (64.2)	22 (88.0)	21 (23.3)	17 (89.5)
	E138APQ	ETR, RPV, NVP	1 (0.4)	0 (0.0)	1 (0.9)	0 (0.0)	0 (0.0)	0 (0.0)
	V179D	EFV, ETR, RPV, NVP	2 (0.7)	0 (0.0)	0 (0.0)	1 (4.0)	0 (0.0)	1 (5.3)
	V179T	ETR by additive NNRTI DRMs	11 (3.9)	1 (2.6)	5 (4.6)	0 (0.0)	4 (4.4)	1 (5.3)
	V179IT	ETR, RPV	2 (0.7)	0 (0.0)	1 (0.9)	0 (0.0)	0 (0.0)	1 (5.3)
	*Y188H	EFV, NVP	1 (0.4)	0 (0.0)	0 (0.0)	0 (0.0)	0 (0.0)	1 (5.3)
*Genotype, n (%)*								
A1		—	220 (78.0)	35 (89.7)	73 (67.0)	22 (88.0)	73 (81.1)	17 (89.5)
CRF 02_AG		—	44 (15.6)	1 (2.6)	29 (26.6)	3 (12.0)	10 (11.1)	1 (5.3)
REC^b^		—	10 (3.5)	2 (5.1)	1 (0.9)	0 (0.0)	7 (7.8)	0 (0.0)
CRF 35_AD		—	7 (2.5)	1 (2.6)	5 (4.6)	0 (0.0)	0 (0.0)	1 (5.3)
C		—	1 (0.4)	0 (0.0)	1 (0.9)	0 (0.0)	0 (0.0)	0 (0.0)

^*^Denotes DRMs included in the World Health Organization 2009 list of mutations for surveillance of transmitted drug-resistant HIV strains.

^a^The Stanford HIVdb was used to associate DRMs with drug susceptibility, where abbreviations refer to PIs: ATV/r, atazanavir/ritonavir; AZT, atazanavir; DOR, doravirine; DRV/r, darunavir/ritonavir; EFV, efavirenz; ETR, etravirine; LPV/r, lopinavir/ritonavir; NNRTIs, non-nucleoside RT inhibitors; NRTIs, nucleoside RT inhibitors; NVP, nevirapine; RPV, rilpivirine.

^b^‘REC’ represents recombinant strains.

DRM E138A was the most common mutation across all cities (58.2%), with almost all sequences from Hyderabad, Larkana, and Quetta containing this mutation (87.2%, 88.0%, and 89.5%, respectively). Karachi sequences had the only reports of DRMs M46I, K219R, and V179T. Despite Peshawar having the smallest percentage (33.3%) of sequences containing any DRMs, these sequences had the most diversity, containing 7 of the 13 DRMs in our analysis, including the only observations of L10F, S68G, K101E, and V106I. The mutation K103N was only reported in two sequences from Peshawar and one sequence from Quetta.

## Discussion

Here, we present HIV DRM from a cross-sectional survey conducted in 2014 among PWID from five cities in Pakistan. Our analysis demonstrated a high prevalence of DRMs associated with NNRTI resistance. The vast majority of participant sequences from Hyderabad (89.7%), Karachi (68.8%), Larkana (88.0%), and Quetta (94.7%) contained at least one DRM. Given that only one (0.6%) study participant reported actively receiving ART, observing DRMs in high frequency among participants suggests that significant HIV transmitted drug resistance is present among PWID in Pakistan.

Over half (58.2%) of PWID in our study harboured E138A, conferring low-level resistance to the NNRTIs etravirine and rilpivirine.^38,39^ Other studies have observed E138A among people living with HIV in Pakistan, reporting varying prevalence in ART-experienced (13%–78%) and ART-naïve (8%–39%) cohorts.^5,27,40^ Perhaps more concerning are the DRMs observed in low prevalence conferring resistance to efavirenz—namely K101E,^41^ K103N,^42,43^ V179D,^44^ and V188H.^45^ Pakistan’s first-line ART regimen for adults is currently tenofovir and lamivudine with dolutegravir, although efavirenz was formerly used in first-line regimens at the time of our study and is currently suggested for use in alternate regimens.^46^ Our results suggest that first-line regimens would be effective treatment for most PWID living with HIV who participated in our study, although this might not be true for previous regimens. While Pakistan has second-line regimens, these drugs are more prone to shortages compared with first-line regimens,^47^ posing challenges to HIV treatment in individuals harbouring resistance to efavirenz prior to the change to dolutegravir,^48^ and enabling transmission of drug-resistant strains.

We observed city of residence and age to be significantly associated with the presence of DRMs. Receiving help at last injection from a professional injector was common among participants with drug resistance (70.3%), and while method at last injection was not significantly associated with drug resistance classification, we did not stratify the analysis by city and injection method to determine its significance for each study city. Professional injectors (or street doctors) are PWID themselves who reuse the same injecting equipment to inject several clients—often younger and inexperienced PWID—daily, for a fee.^49–51^ This fee can be something known as ‘scale’, a blood-drug mixture collected by the professional injector by double-pumping the syringe, which is then sold to other PWID for less than the regular drug,^22,29,52^ allowing for efficient transmission of drug-resistant HIV. Using help from professional injectors has previously been reported to be more prevalent in Karachi (53.7%) compared with Peshawar (25.2%) and Quetta (19.6%).^50^ Differences in DRM prevalence among cities might also be partially explained by varying rates of heroin injection, observed to be high in Karachi and Quetta (96.9% and 81.1% respectively).^50^ Heroin has been associated with high-risk injection practices, such as needle sharing and ‘backloading’ (also known as ‘flushblood’ or ‘jerking’), where heroin is mixed with blood and shared amongst injectors,^6^ facilitating transmission of resistant strains among PWID.

In this study, we report that the spread of drug-resistant HIV is likely being fuelled by the lack of ART coverage among PWID participants, permitting high viral loads which enable efficient HIV transmission. Our findings stress the lack of ART coverage among PWID in Pakistan, known to still be an ongoing issue and major obstacle to HIV treatment in the country.^53^ The low ART coverage observed in our study is likely due in part to the lack of participants’ awareness that they are living with HIV overall, as we observed nearly all (88.7%) PWID in our study to have never undergone HIV testing. Awareness of HIV status among people in Pakistan is known to be low (21%), with people living with HIV actively receiving ART being even lower (16%) as of 2024.^2^ This is further compounded by high rates of loss-to-follow-up during treatment. In 2018, almost 37% of people living with HIV in Punjab and Sindh receiving ART were lost to follow-up,^53^ which may be partially explained by social stigma experienced in healthcare settings.^54^ Concerns regarding confidentiality and disclosure of patient HIV status without consent have also been reported at treatment centres, which may discourage people living with HIV from seeking and/or continuing ART treatment.^53,55^

The criminalized status of PWID makes it challenging for this population to access HIV programs initially and consistently thereafter across global contexts,^56^ including Pakistan,^6^ where interruptions in care and fluctuating adherence to ART may promote the emergence of DRMs. This challenge is compounded locally by the requirement that PWID undergo detoxification prior to initiating ART, further delaying timely treatment.^11^ In addition, Pakistan lacks a national opioid substitution therapy program, a key component of effective HIV treatment and prevention that remains illegal.^7,11^

Given these intersecting structural and systemic barriers, it has been suggested that HIV prevention and treatment efforts in Pakistan could be made more effective and contextually appropriate through service decentralization, improved health system capacity, and community-based service delivery models to reduce burden on existing infrastructure and improve retention in care.^53,57^ Despite the availability of harm reduction services, national HIV surveillance conducted in 2016–2017 found that fewer than one-quarter (22.4%) of PWID had ever received new syringes from a service delivery program.^58^ The same survey also reported a high prevalence of illiteracy (40.1%) among PWID, which poses additional challenges for educational interventions aimed at HIV prevention, treatment adherence, and regular follow-up at treatment centres.^53,58^

We acknowledge several limitations in our study. First, the data were collected several years ago, which may limit conclusions about the current distribution of HIV drug resistance in Pakistan. However, our primary objective was to highlight the persistent and underrecognized issue of transmitted drug resistance, not to provide current prevalence estimates *per se*. Given the absence of recent transmitted drug resistance data among PWID in Pakistan, historical insights remain valuable for identifying gaps and guiding future interventions. Without such context, it is difficult to appreciate how drug resistance may be evolving in settings with suboptimal treatment coverage and surveillance. Second, varying sample sizes across cities may affect the generalizability of observed drug resistance patterns, particularly among cities with smaller representation. Third, the cross-sectional design limits causal inference and prevents evaluation of temporal trends. Incomplete ART histories also prevented us from fully distinguishing between acquired and transmitted DRMs. However, ART uptake and coverage have historically been, and continue to be, notably low among PWID in Pakistan; therefore, our overall conclusions would likely remain unchanged, even with comprehensive ART records. Fourth, self-reported behavioural data may be subject to social desirability bias, especially for sensitive topics such as drug use and HIV status. Lastly, our in-house genotyping assay did not encompass the integrase gene, limiting detection of dolutegravir-specific DRMs. While future studies should incorporate integrase sequencing, this gene was of limited relevance at the time of sample collection, as integrase inhibitors were unlikely to be part of Pakistan's treatment guidelines during that period.

Despite these limitations, the findings of this study remain relevant given the ongoing challenges in the national HIV response, including persistently low ART coverage, suboptimal adherence, and limited access to genotypic resistance testing in Pakistan.^2,14,26^ Through sequencing and genotyping, we identified DRMs amongst PWID in Pakistan conferring low-level resistance to etravirine, rilpivirine, and efavirenz. While our results suggest the majority of PWID in our study would be susceptible to current first-line ART regimens, perhaps more concerning is their lack of awareness of HIV-positive status and extremely limited access to harm reduction and treatment. Although there remains a shortage of recent DRM studies conducted among PWID in Pakistan, findings from more recent studies conducted among the broader pediatric and adult populations highlight that drug resistance remains an urgent concern in the country.^27,40^ Given the implementation of drug resistance genotyping prior to ART in this particular setting is unlikely (as is the case for many low-resource settings), our study highlights the need for sustained and ongoing surveillance of DRMs to monitor the emergence and spread of HIV drug resistance, inform treatment strategies, and ensure that ART regimens remain effective.
